# JNKi- and DAC-programmed mesenchymal stem/stromal cells from hESCs facilitate hematopoiesis and alleviate hind limb ischemia

**DOI:** 10.1186/s13287-019-1302-1

**Published:** 2019-06-24

**Authors:** Yimeng Wei, Huixing Hou, Leisheng Zhang, Nianhuan Zhao, Chengwen Li, Jiali Huo, Ying Liu, Wenxia Zhang, Zongjin Li, Dengke Liu, Zhibo Han, Lei Zhang, Baoquan Song, Ying Chi, Zhongchao Han

**Affiliations:** 1 State Key Laboratory of Experimental Hematology, Institute of Hematology & Blood Diseases Hospital, Chinese Academy of Medical Sciences & Peking Union Medical College, 288 Nanjing Road, Tianjin, 300020 China; 2 Department of Gastroenterology and Hepatology, General Hospital, Tianjin Medical University, Tianjin, 300052 China; 3 School of Medicine, Nankai University, Tianjin, 300071 China; 4 The Postdoctoral Research Station, College of Life Science, Nankai University, Tianjin, 300071 China; 5 The Enterprise Postdoctoral Working Station, Tianjin Chase Sun Pharmaceutical Co., Ltd., Tianjin, 301700 China; 6 Precision Medicine Division, Health-Biotech (Tianjin) Stem Cell Research Institute Co., Ltd., Tianjin, 301700 China; 7 Jiangxi Research Center of Stem Cell Engineering, Jiangxi Health-Biotech Stem Cell Technology Co., Ltd., Shangrao, 334000 China; 8 Jiangsu Institute of Hematology, The First Affiliated Hospital of Soochow University, Suzhou, 215006 China

**Keywords:** Programming, MSCs, Hematopoiesis, Hind limb ischemia, hESCs

## Abstract

**Background:**

Mesenchymal stem/stromal cells (MSCs) derived from human embryonic stem cells (hESCs) are attractive for their hematopoietic-supporting or potential therapeutic effects. However, procedures for high-effective and scalable generation of MSCs from hESCs within 2 weeks are still unestablished, which also hinder the development and mechanism study of mesengenesis.

**Methods:**

In this study, we aimed to establish a strategy for programming hESC differentiation into MSCs by practicing small-scale chemical compound screening. Then, we used flow cytometry, multi-lineage differentiation, and karyotype analyses to investigate the biological phenotypes of the derived hESC-MSCs. Also, to explore whether the derived cells had hematopoietic-supporting ability in vitro, we carried out the cobblestone formation and megakaryocytic differentiation experiments. To further evaluate the function of hESC-MSCs in vivo, we transplanted the cells into a mouse model with hind limb ischemia.

**Results:**

By simultaneous treatments with a JAK/STAT antagonist and a DNA methylation inhibitor, the efficiency of generating hESCs into CD73^+^ hESC-MPCs could reach 60% within 7 days. The derived cells further matured into hESC-MSCs, with comparable characteristics to those of adult MSCs in terms of surface markers, normal karyotype, and the potential for adipogenic, osteogenic, and chondrogenic differentiation. Functionally, hESC-MSCs had hematopoietic-supporting effects in vitro and could notably relieve symptoms of hind limb ischemia.

**Conclusions:**

In the study, we established a high-efficient procedure for large-scale generation of MSCs from hESCs, which would be of great help for genesis and mechanism studies of MSCs. Meanwhile, the derived cells provide an alternative for translational clinical research.

**Electronic supplementary material:**

The online version of this article (10.1186/s13287-019-1302-1) contains supplementary material, which is available to authorized users.

## Background

Owning to the self-renewal capacity, multi-lineage differentiation potential, immunosuppressive property, and paracrine mechanism, mesenchymal stem/stromal cells (MSCs) have been demonstrated a promising source for regenerative medicine [1]. To date, adult tissue-derived MSCs are most commonly used for more than 300 clinical trials [2, 3], especially for cell-based therapies in amounts of disease treatments, including diabetes, hypertension, arthritis, liver cirrhosis, autoimmune disorders, wound repair, myocardial ischemia, and hematopoietic reconstitution [4–11]. However, these adult MSCs have several disadvantages and limitations [12], including the instability and heterogeneity of the source, and decreased therapeutic efficacy after ex vivo expansion [13]. Additionally, the long-term proliferation property and multi-lineage differentiation capacity of adult MSCs isolated from aged donors will dramatically decline as well [12]. To overcome these major shortcomings, there is an imperious demand for alternative sources of MSCs.

Human embryonic stem cells (hESCs), together with human induced pluripotent stem cells (hiPSCs), belong to human pluripotent stem cells (hPSCs) [14, 15]. These cell sources have incomparable self-renewal capacity and the potential to differentiate into more than 200 cell types including MSCs [16, 17]. For decades, considerable studies have indicated the potential of hESCs to be a perfect alternative of MSCs by utilizing animal models of human disease [12, 13, 18]. On one hand, hESC-derived MSCs show similarities in biological phenotype and comparable functions with adult MSCs, but have superiorities in long-term proliferation and homologous quality [18–20]. On the other hand, the studies on hESC mesenchymal differentiation will help investigate the developmental process and molecular mechanism of MSCs and provide potential sources for patient-specific autologous therapy [13, 21, 22].

Over the last decade, there have been three major sorts of approaches for MSC induction from hESCs, including the three-dimensional embryoid body (EB) model, co-culture model, and monolayer induction model [12, 23–25]. During the years, although a number of mesenchymal progenitor cells or mature MSCs were acquired from hESCs, most of the current procedures have common disadvantages, including low efficiency and multifarious manipulations such as scraping, serial passages, handpicking, or enrichment by cell sorting [18]. More importantly, due to the absence of high-efficient differentiation procedures, the developmental process and underlying mechanism of mesengenesis are largely unknown [13, 21]. Contrarily, although it is still time-consuming and low-efficient to obtain amounts of mature and homologous MSCs, encouraging advances on various disease treatments have been achieved by MSC transplantation, including multiple sclerosis, graft-versus-host-disease (GVHD), hind limb ischemia, DSS-induced colitis, bone marrow failure, and experimental inflammatory bowel disease [13, 18, 26]. Thus, for practical translation of MSCs in the future, it is of great importance for robust and functional MSC generation from hESCs and to dissect the complex orchestra of signals and transcription factors.

Recently, we have reported a programming strategy for high-efficient generation of MSCs from hPSCs with MSX2 ectopic expression and a cocktail of four soluble molecule addition [13], yet the protocol could not be used for therapeutic purposes. Thus, it is urgent to develop a more convenient procedure for MSC generation without gene editing. In this study, by performing a small-scale screening of chemical compounds and growth factors, we found with the aid of a JAK/STAT signaling pathway antagonist (OICR-9429, short for JNKi) and a DNA methyltransferase inhibitor (decitabine, short for DAC), 60% of hESCs automatically differentiated into hESC-MPCs within 1 week in MSC culture media. By passaging, the derived cells matured into hESC-MSCs in another 1 week and satisfied the criteria of MSCs in biomarker expression, normal karyotype, and multi-lineage differentiation capacity, but with better long-term proliferation ability than adult MSCs. Furthermore, the hESC-MSCs had hematopoietic-supporting potential in vitro and showed therapeutic effect in alleviating hind limb ischemia in vivo comparable with those of adult MSCs.

## Methods

### hESC culture and hESC-MSC differentiation

hESC lines (H1 hESCs, WiCell Research Institute) were cultured as we previously reported [13, 14]. Briefly, the cells were seeded on 12-well plate (Corning) coated with matrigel (BD Bioscience) in mTeSR1 medium (Stem Cell Technologies) at 5% CO_2_ and 37 °C. For MSC induction, hESCs were split into single cells with Accutase (Gibco), then seeded in mTeSR1 medium with Y27632 (10 nM) (Sigma) addition on a growth factor reduced gel (GFR, Stem Cell Technologies)-coated 6-well plate at a density of 1.3 × 10^4^ cells/mL. After 2 days, the spent medium was replaced by DMEM/F12 basal medium (Hyclone) containing 5% fetal bovine serum (Gibco), 1% penicillin-streptomycin (Gibco), 1% l-glutamine (Gibco), and 10 nM small molecule (Targetmol) from day 0 to day 7; then, cells were transferred into an adherent culture plate in DMEM/F-12 medium supplemented with 10% FBS, 1% penicillin-streptomycin (Gibco), 1% l-glutamine (Gibco), and 5 nM Y27632 (Sigma). Cells were cultured for another 7 days, and media were changed every 2 days in the entire process.

### Isolation of CD34^+^ HSCs from the human umbilical cord blood (UCB-CD34^+^ HSCs)

Human umbilical blood mononuclear cells were isolated from blood of healthy donors by standard Ficoll density gradient centrifugation as we previously reported [15]. The isolated cells were used for UCB-CD34^+^ HSC enrichment by magnetic cell sorting. The enriched UCB-CD34^+^ HSCs were further cultured in RPMI-1640 basal medium (Hyclone) supplemented with 10% FBS in 37 °C and 5% CO_2_ incubator.

### Enrichment and purification of platelets

Enrichment and purification of platelets were performed by centrifugation at 800×*g* for 10 min as we previously reported [15]. Before further morphological and functional assay, platelets were resuspended in 1 × CSG buffer containing 1 μM prostaglandin E1 (PGE1, Sigma) and maintained at room temperature.

### Aggregation test of platelets

To analyze the aggregation potential of the platelets, peripheral blood platelets labeled with Calcein-AM (Invitrogen) were mixed with the mouse anti-β1-tubulin (GE Healthcare)-labeled platelets. The platelet aggregates were stained with 594-conjugated donkey anti-mouse IgG (Invitrogen) after agonist incubation. Immunofluorescent images of platelets were observed under a confocal laser scanning microscope (Leica).

### Megakaryocytic differentiation and platelet generation

For megakaryocytic differentiation and platelet generation, the purified umbilical cord blood CD34^+^ (UCB-CD34^+^) cells were co-cultured with hESC-MSCs or hBM-MSCs for 9 days. UCB-CD34^+^ cells were co-cultured at a density of 1 × 10^5^ cells/mL in the hematopoietic medium with the presence of TPO (20 ng/mL), SCF (20 ng/mL), IL-3 (10 ng/ml), IL-6 (10 ng/ml), IL-9 (10 ng/ml), IL-11(10 ng/ml), and Y27632 (10 nM). The spent medium was replaced every 3 days.

### Animals and mouse hind limb ischemia model

BALB/c mice (female, 8–10 weeks, 18–22 g) in our research were purchased and approved (approval no. KT2016011-EC-1) for use by the Peking Union Medical College Institutional Animal Care and Use Committee (license no. SCXK & SYXK 2005-0001, Tianjin). BALB/c mice were intraperitoneally anesthetized with 350 mg/kg chloral hydrate (Sigma). The procedure for establishing the hind limb ischemia model was described previously [27]. Briefly, ligation and excision were undergone on the proximal and distal end of the femoral artery after dissection from the femoral vein and nerve. Post surgery, mice were randomly divided into two groups (+PBS, +hESC-MSC groups), and 1 × PBS or 1 × 10^6^ hESC-MSC suspension at a 100-μL volume was intramuscularly injected into ischemia hind limbs, respectively. Normal BALB/c mice without surgery were served as controls (NT).

### Assessment of limb function and ischemia damages

At day 14 or day 28 post operation, limb function scoring was performed by a semi-quantitative assessment using a modified clinical score as we previously reported [27] (0 = toe flexion, 1 = plantar flexion, 2 = no dragging but no plantar flexion, 3 = foot dragging). The ischemia damages were also assessed (0 = no difference from the normal hind limb, 1 = mild discoloration, 2 = moderate/severe discoloration, 3 = necrosis, and 4 = any amputation).

### Histological analysis

On day 28 of the ischemia model, the adductor muscle samples from euthanized animals of each group were collected, then fixed with 10% formaldehyde overnight, and embedded in paraffin as we previously reported [27]. To evaluate fiber degeneration and apoptosis, muscle tissues were stained with hematoxylin and eosin (H&E). To estimate the degree of muscle fibrosis, Masson staining was performed on sample tissues. All images were observed and captured under × 200 magnification.

### Population doubling assay

To estimate the proliferation capacity of MSCs, 1 × 10^4^ hESC-MSCs and hBM-MSCs were respectively collected and seeded into 3-cm^2^ plates as we previously reported [13, 15]. Cells were cultured for 7 days with replacement of culture medium every 3.5 days. Population doubling number (PDN) was quantified using the formula PDN = log*N*/*N*_0_ × 3.31, and population doubling time (PDT) was calculated using the formula PDT = (*t* − *t*_0_)∙log2/log(*N* − *N*_0_), where *N*_0_ is the initial number of seeded cells is the number of harvested cells and *t* − *t*_0_ is the time of cell culture.

### Flow cytometry analysis

For flow cytometry analysis of the phenotype of MPCs or hESC-MSCs, cells were collected and stained with antibodies in dark for 30 min as we previously reported [13, 15]. The antibodies used in this study were PE-conjugated CD29, PE-conjugated CD31, APC-conjugated CD34, FITC-conjugated CD45, APC-conjugated CD41a, PE-conjugated CD42b, PE-conjugated CD44 (BD Pharmigen), APC-conjugated CD105 (BioLegend), PE-conjugated CD106, PerCP-cy5.5-conjugated CD73, and FITC-conjugated CD90 (eBioscience). Data was acquired by Canto II flow cytometer (BD Biosciences) and analyzed with FlowJo 7.6 software (San Carlos, CA, USA). The antibody list is available in Additional file 10: Additional information.

### Immunofluorescence

The expression level of OCT4 and CD73 was examined according to the manufacturer’s instructions. Spent culture medium of hESCs or hESC-MPCs was discarded, then the cells were washed with PBS for twice, fixed with 4% formaldehyde for 15–20 min, and blocked with blocking reagent for 1 h. For the assessment of OCT4 expression, hESCs and hESC-MPCs were permeabilizated with 0.2% Triton™ X-100 for 1 h, then cells were stained with rabbit-anti-human OCT4 for 12 h and stained with donkey anti-rabbit Alexa Fluor 488-conjugated secondary antibody for an hour [14]. For the assessment of CD73 expression, cells were stained with mouse anti-human CD73 for 12 h and then stained with Alexa Fluor 594-conjugated secondary antibody (donkey anti-mouse) for 1 h. DAPI was used for nucleus staining.

### Multi-lineage differentiation assay

For induction of adipogenic differentiation, hESC-MSCs and hBM-MSCs were seeded at a density of 2 × 10^4^ cells/cm^2^ and cultured in adipogenic differentiation medium (MesenCult™ Adipogenic Differentiation Kit, Stem Cell Technologies) and the medium was changed every 3 days. On day 21 of differentiation, cell culture was discontinued for histochemical staining and total RNA extraction. Oil red O staining was performed to detect fat droplets in differentiated cells. For induction of osteogenic differentiation, hESC-MSCs and hBM-MSCs were seeded at a density of 5 × 10^3^ cells/cm^2^ and cultured in osteogenic differentiation medium (MesenCult™ Osteogenic Differentiation Kit, Stem Cell Technologies) and the medium was changed every 3 days. After 28 days of differentiation, cell culture was stopped for histochemical staining and total RNA extraction. Alizarin red staining was used to identify calcium deposition. For induction of chondrogenic differentiation, hESC-MSCs and hBM-MSCs were seeded at a density of 5 × 10^3^ cells/cm^2^ and cultured in chondrogenic differentiation medium (MesenCult™-ACF Chondrogenic Differentiation Kit, Stem Cell Technologies) and the medium was changed every 3 days. Cell culture was fixed for histochemical staining and total RNA extraction on day 28 of differentiation. Alcian blue staining was performed for determination of sulfated proteoglycans within chondrogenic differentiated cells. Images of differentiated cells were photographed under a Nikon ElipseTi-U microscope (Nikon, Tokyo, Japan).

### Quantitative real-time PCR

Total RNA extraction was performed using the E.Z.N.A Total RNA Kit I (OMEGA, Norcross, GA, USA), and cDNA synthesis was performed using the TransScript Fly First-Strand cDNA Synthesis SuperMix (Transgen Biotech, China). All procedures were implemented according to the manufacturer’s instructions. Quantitative real-time PCR was performed using the QuantStudio 6 Flex Real-Time PCR System (Applied Biosystems) and the SYBR™ Green PCR Master Mix (Applied Biosystems). All samples were analyzed in triplicate. The expression of *ACTIN*, *POU5F1*, *SOX2*, *NANOG*, *T*, *GATA2*, *PAX6*, *TROP2*, *NT5E*, *ENG*, *VIM*, *FN1*, *ADIPOQ*, *PPAR-γ*, *RUNX2*, *BGLAP*, *ACAN*, *SOX9*, *TPO*, *SCF*, *IL-3*, *IL-6*, *IL-9*, *IL-11*, *EPO*, *GATA1*, *FLI-1*, *RUNX1*, *FOG-1*, *NF-E2*, *ITGB3*, *VEGFA*, *VEGFB*, *ANG1*, and *ANG2* genes were detected as we previously reported. The premier list is available in Additional file 10: Additional information.

### Colony-forming unit (CFU) assay

Purified umbilical cord blood CD34^+^ cells were co-cultured with hESC-MSCs or hBM-MSCs for 2 weeks [15]. To estimate the effects of MSCs on CFU growth in vitro, the co-cultured UCB-CD34^+^ cells were collected and seeded on a sterile 24-well plate and incubated in methylcellulose medium (Stem Cell). Then, numbers of colonies were calculated. Each group’s experiments were implemented in triplicates.

### Cobblestone formation

Confluence of UCB-CD34^+^ cells and hESC-MSCs or hBM-MSCs were cultured on a 35-mm dish at 37 °C, 5% CO_2_ in MSC culture medium supplied with 10% horse serum, and spent media were replaced every 3 days as previously reported [28]. Cobblestone area started to emerge on day 5 of confluence and can be observed on day 7. Small and compact clusters of more than 5 cells were considered to be cobblestone area-forming cells. All images were observed and photographed under a phase contrast microscope.

### Karyotype analysis

To monitor the genomic stability of hESC-MSCs or hBM-MSCs, karyotype analysis was performed using a G-banding technique, which the procedure was described previously [21]. Chromosomes of cells were observed and captured with × 200 magnification under an Olympus DP71 microscope (Tokyo, Japan).

### Statistical analysis

Data of two unpaired groups were analyzed with unpaired *t* test, and data of multiple unpaired groups were analyzed with one-way ANOVA with Tukey’s post hoc test. Statistical analysis was performed by Prism 6.0 (GraphPad Software, San Diego, CA, USA). Statistical significance was determined when *P* value was less than 0.05 as we previously reported [13–15].

## Results

### JNKi and DAC initiate generation of hESC-MPCs from hESCs

We recently reported that with the aid of *MSX2*, a transcription factor and a cocktail of four chemical compounds, nearly all hPSCs were programmed into functional MSCs in 1 week [13]. The procedure demonstrated to be more rapid and cost-effective than the previously described methods involving the EB induction, co-culture, or monolayer model. However, the derived MSCs were not sufficient for therapeutic purposes due to viral integration of transgenes.

**Fig. 1:**
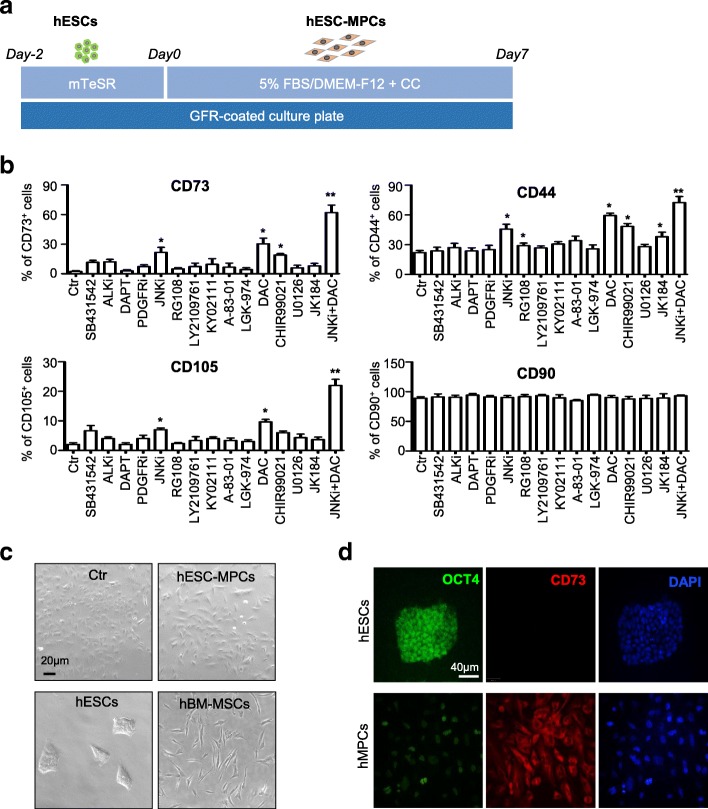
JNKi and DAC initiate generation of hESC-MPCs. **a** Schematic of mesenchymal differentiation from hESCs to hESC-MPCs. Single H1 hESCs were seeded on GFR-coated 6-well plate for 2 days, then the mTeSR medium was changed into DMEM/F12 media supplied with 5% FBS and/or chemical compound (CC) for a week. **b** Flow cytometry (FCM) analysis of MSC marker of hESC-derived cells cultured in 5% FBS/DMEM/F12 ± 10 nM chemical compounds for 7 days (mean ± SEM, *N* = 3). *P < 0.05;**P < 0.01. **c** Morphology of H1 hESCs cultured in 5% FBS/DMEM/F12 with (hESC-MPCs) or without (Ctr) JNKi and DAC addition for 7 days, undifferentiated hESCs (hESCs), and hBM-MSCs (scale bar = 20 μm). **d** Representative immunofluorescence images display the expression of OCT4 (in green) and CD73 (in red) in hESCs and hESC-MPCs (scale bar = 40 μm). The nuclei (in blue) were labeled with DAPI

Thus, in this study, we conducted a small-scale screening of chemical compounds and growth factors to derive MSCs from hESCs under MSC culture media as we previously reported with modification [13] (Fig. 1a). At day 7 of the induction, we measured the proportion of hESC-derived mesenchymal progenitor cells (hESC-MPCs) by flow cytometry as reported [13]. Compared with the control group (short for “Ctr” thereafter), we found that the addition of OICR-9429 (a JAK signal and histone methyltransferase inhibitor), RG108, DAC, CHIR99021, or JK184 facilitated the generation of CD73^+^ or CD44^+^ cells, but only JNKi and DAC promoted the proportion of CD105^+^ cells as well (Fig. 1b, Additional file 1: Figure S1a). These data led us to speculate sole JAK/STAT antagonist and a DNA methylation inhibitor might make the same results. Consistently, we found that the addition of ruxolitinib (a JAK/STAT antagonist) or AZA (DNA methylation inhibitor) also facilitated the generation of CD73^+^ and CD105^+^ cells, but lower than the JNKi or DAC group, respectively (Additional file 1: Figure S1b). Then, we combined JNKi with DAC to induce MSC generation, nearly 60% hESCs were derived into CD73^+^ cells within 7 days without CD31, CD34, and CD45 expression (Fig. 1b, Additional file 1: Figure S1c). Consistently, the cells showed elongated and spindle-like shape as adult bone marrow MSCs (hBM-MSCs), but distinguished morphological changes to the undifferentiated hESCs or the Ctr cells (Fig. 1c). Furthermore, with immunofluorescence staining, we confirmed the upregulation of CD73, a MSC marker, and the sharp decrease of OCT4, a pluripotency marker, in hESC-MPCs (Fig. 1d). Herein, by screening a small scale of chemical compounds, we demonstrated that simultaneous treatments with JNKi and DAC could high-efficiently programmed hESCs into hESC-MPCs.

### High-efficiency generation of homogeneous hESC-MSCs from hESC-MPCs

**Fig. 2:**
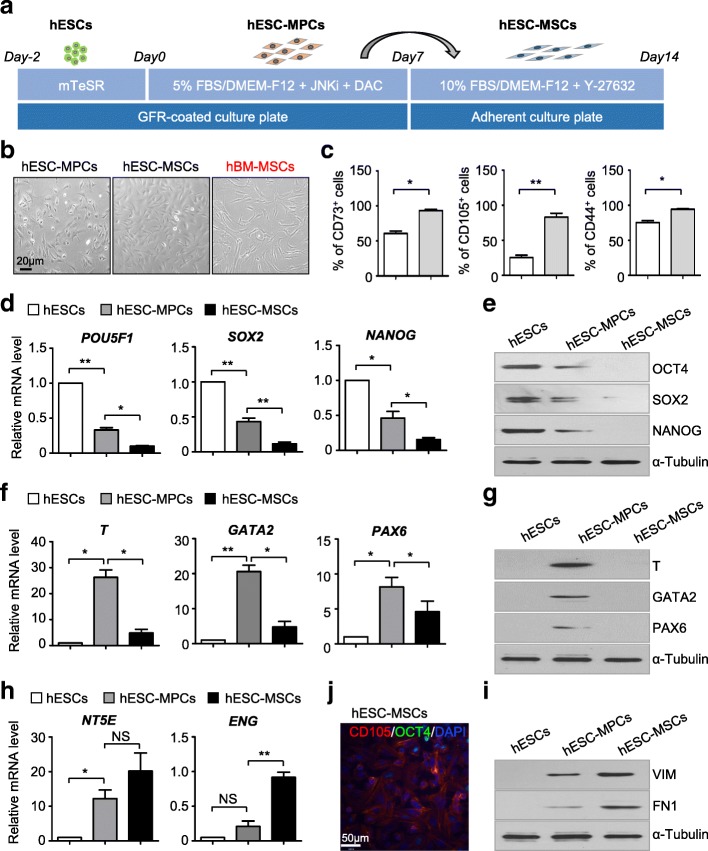
High-efficiency generation of homogeneous hESC-MSCs. **a** Schematic of mesenchymal differentiation from hESCs to hESC-MSCs. Briefly, single H1 hESCs were seeded on GFR-coated 6-well plate for 2 days, then the mTeSR medium was changed into DMEF/F12 media supplied with 5% FBS, JNKi, and DAC for a week. To generate more mature MSCs (hESC-MSCs), hESC-MPCs were passaged onto adherent culture plate, and the medium was changed into DMEF/F12 media supplied with 10% FBS and 10 nM Y-27632 for another week. **b** Morphology of derived hESC-MSCs and hBM-MSCs (scale bar = 20 μm). **c** Flow cytometry (FCM) analysis showing the percentage of CD73^+^, CD105^+^, and CD44^+^ cells in hESC-MPCs and hESC-MSCs. Data are shown as mean ± SEM (*n* = 3). *P < 0.05; **P < 0.01; NS, not significant. **d** qRT-PCR analysis of pluripotency markers (*POU5F1*, *SOX2*, *NANOG*) in undifferentiated hESCs, hESC-MPCs, and hESC-MSCs. Data are shown as mean ± SEM (*n* = 3). *P < 0.05; **P < 0.01. **e** Western blotting analysis of pluripotency markers (OCT4, SOX2, NANOG) in undifferentiated hESCs, hESC-MPCs, and hESC-MSCs. α-Tubulin was used as a loading control. **f** qRT-PCR analysis of germ layer-associated genes (*T*, *GATA2*, *PAX6*) in undifferentiated hESCs, hESC-MPCs, and hESC-MSCs. Data are shown as mean ± SEM (*n* = 3). *P < 0.05; **P < 0.01; NS, not significant. **g** Western blotting analysis of germ layer-associated genes (T, GATA2, PAX6) in undifferentiated hESCs, hESC-MPCs, and hESC-MSCs. α-Tubulin was used as a loading control. **h** qRT-PCR analysis of MSC-associated genes (*NT5E*, *ENG*) in undifferentiated hESCs, hESC-MPCs, and hESC-MSCs. Data are shown as mean ± SEM (*n* = 3). *P < 0.05; **P < 0.01; NS, not significant. All values are normalized to the hESC group (= 1). **i** Western blotting analysis of MSC-associated genes (VIM. FN1) in undifferentiated hESCs, hESC-MPCs, and hESC-MSCs. α-Tubulin was used as a loading control. **j** The representative immunofluorescence image displays the expression of OCT4 (in green) and CD105 (in red) in hESCs (scale bar = 50 μm). The nuclei (in blue) were labeled with DAPI

To further develop the hESC-MPCs into more mature MSCs, we passaged the cells into MSC culture medium with 10% FBS and 5 nM Y-27632 addition for one more week according to the previous reports [18] (Fig. 2a). Strikingly, the derived cells showed more typical elongated and spindle-like shape (Fig. 2b). Furthermore, by surface marker detection of the derived cells, we found nearly all cells turned into CD73^+^, CD105^+^, and CD44^+^ cells, much similar to hBM-MSCs but higher than hESC-MPCs (Additional file 2: Figure S2a). Thus, we denoted the more mature and homogeneous cells as hESC-MSCs (Fig. 2c). However, different from hBM-MSCs, hESC-MSCs expressed a lower level of CD106 (Additional file 2: Figure S2b). To further characterize the hESC-MSCs in the molecular level, we measured the expression of typical pluripotency markers, germ layer markers, and MSC markers among the undifferentiated hESCs, hESC-MPCs, and hESC-MSCs by qRT-PCR and western blotting. Consistently, compared to the other groups, pluripotent markers including *OCT4*, *SOX2*, and *NANOG* were further decreased in hESC-MSCs, indicating hESC differentiation (Fig. 2d, e). Meanwhile, by examining the expression pattern of genes indicating germ layers, we found the significant upregulation of mesodermal, endodermal, and ectodermal marker genes, *T*, *GATA2*, and *PAX6*, in hESC-MPCs and subsequent downregulation in hESC-MSCs, except for the trophectodermal marker, *TROP2* [12] (Fig. 2f, g, Additional file 2: Figure S2c-S2d). Furthermore, we found that the expression of typical MSC markers such as *NT5E* (also known as *CD73*), *ENG* (also known as *CD105*), *VIM*, and *FN1* were elevated progressively and dramatically in hESC-MSCs (Fig. 2h, i, Additional file 2: Figure S2e). Moreover, by practicing immunofluorescence staining, we confirmed the expression of CD105, a MSC marker, and the sharp decrease of OCT4, a pluripotency marker, in hESC-MSCs (Fig. 2j, Additional file 2: Figure S2f). In summary, we have established a two-step procedure for high-efficiency generation of mature and homogeneous hESC-MSCs within 2 weeks.

### The hESC-MSCs exhibit signatures of adult MSCs but with superiority in long-term proliferation

**Fig. 3:**
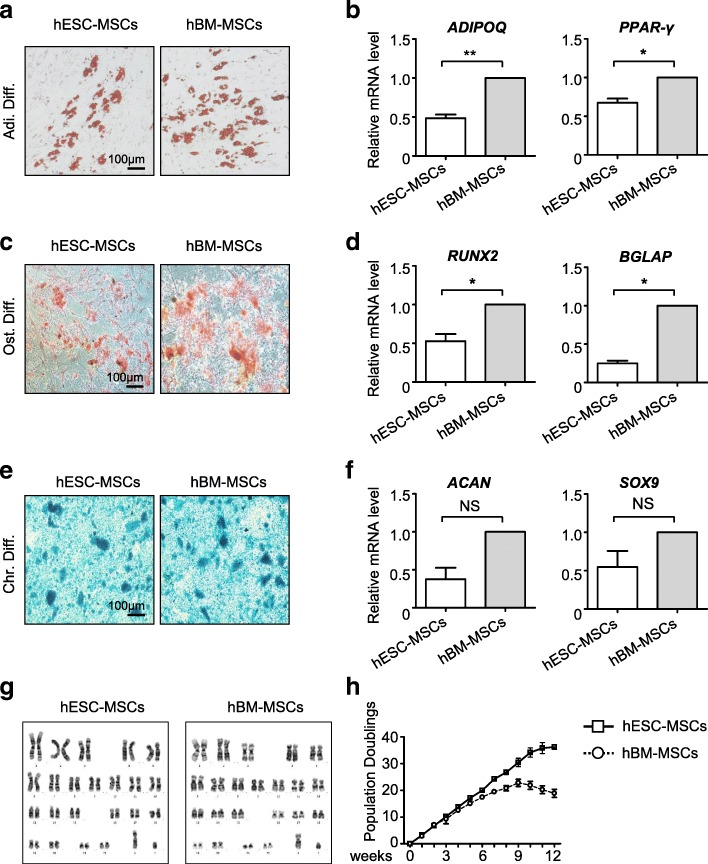
The hESC-MSCs exhibit signatures of adult MSCs but superiority in long-term proliferation. **a** Adipogenic differentiation potential of hESC-MSCs and hBM-MSCs was identified by Oil red O staining (scale bar = 100 μm). **b** qRT-PCR analysis of the adipogenic markers (*ADIPOQ*, *PPAR-γ*) in hESC-MSCs and hBM-MSCs. Data are shown as mean ± SEM (*n* = 3). *P < 0.05; **P < 0.01. All values are normalized to the hBM-MSC group (= 1). **c** Osteogenic differentiation potential of hESC-MSCs and hBM-MSCs was identified by Alizarin red staining (scale bar = 100 μm). **d** qRT-PCR analysis of the osteogenic markers (*RUNX2*, *BGLAP*) in hESC-MSCs and hBM-MSCs. Data are shown as mean ± SEM (*n* = 3). *P < 0.05. All values are normalized to the hBM-MSC group (= 1). **e** Chondrogenic differentiation potential of hESC-MSCs and hBM-MSCs was identified by Alcian blue staining (scale bar = 100 μm). **f** qRT-PCR analysis of the chondrogenic markers (*ACAN*, *SOX9*) in hESC-MSCs and hBM-MSCs. Data are shown as mean ± SEM (*n* = 3). NS, not significant. All values are normalized to the hBM-MSC group (= 1). **g** Karyotype analysis of hESC-MSCs and hBM-MSCs with G-banded chromosome experiment. **h** Expansion potential of hESC-MSCs and hBM-MSCs for 12 passages by population doubling assay (Pd). Data are shown as mean ± SEM (*N* = 3)

To clarify whether the derived hESC-MSCs were functionally mature as adult MSCs (hBM-MSCs), we initially examined the multi-lineage differentiation potential of the derived hESC-MSCs. After 3 weeks of adipogenic differentiation, both of the hESC-MSCs and hBM-MSCs were positive for Oil Red O staining (Fig. 3a). However, in the expression analysis of the adipogenic markers, *ADIPOQ* and *PPAR-γ*, we found the hESC-MSCs were lower than hBM-MSCs (Fig. 3b). Similarly, the osteogenic differentiation potential of the hESC-MSCs was also weaker than hBM-MSCs, confirmed by Alizarin Red staining and qRT-PCR analyses of the osteogenic markers, *RUNX2* and *BGLAP* (Fig. 3c, d). Also, we found there was no significant difference in chondrogenic differentiation between the two groups, identified by Alcian blue staining and the expression of chondrogenic markers, *ACAN* and *SOX9* (Fig. 3e, f). However, comparing hESC-MSCs with another adult MSCs (human adipose-derived MSCs, hAD-MSCs), we found the osteogenic and chondrogenic differentiation potentials of hESC-MSCs were higher than those of hAD-MSCs, while the adipogenic potential of hESC-MSCs was lower (Additional file 3: Figure S3a-S3b). Additionally, to assess the tissue differentiation potential, we transplanted MSCs into immunodeficient mice as reported [13]. Like hBM-MSCs, hESC-MSCs were capable of forming bone in vivo (Additional file 3: Figure S3c). Furthermore, by performing G-banded chromosome studies, we confirmed the derived hESC-MSCs exhibited normal karyotype without gross abnormalities at the genomic level as hBM-MSCs (Fig. 3g). Most importantly, we and other researchers previously reported that hESC- or hiPSC-derived MSCs have unlimited proliferation potential [12, 13]. Thus, in this study, by practicing the populating doubling (Pd) assay, we confirmed the derived hESC-MSCs could also be cultivated consecutively for 12 passages (Fig. 3h). In summary, the high-efficiency generation of large-scale and mature MSCs could be accomplished in 2 weeks from hESCs.

### The hESC-MSCs show comparable hematopoietic-supporting potential as hBM-MSCs in vitro

**Fig. 4:**
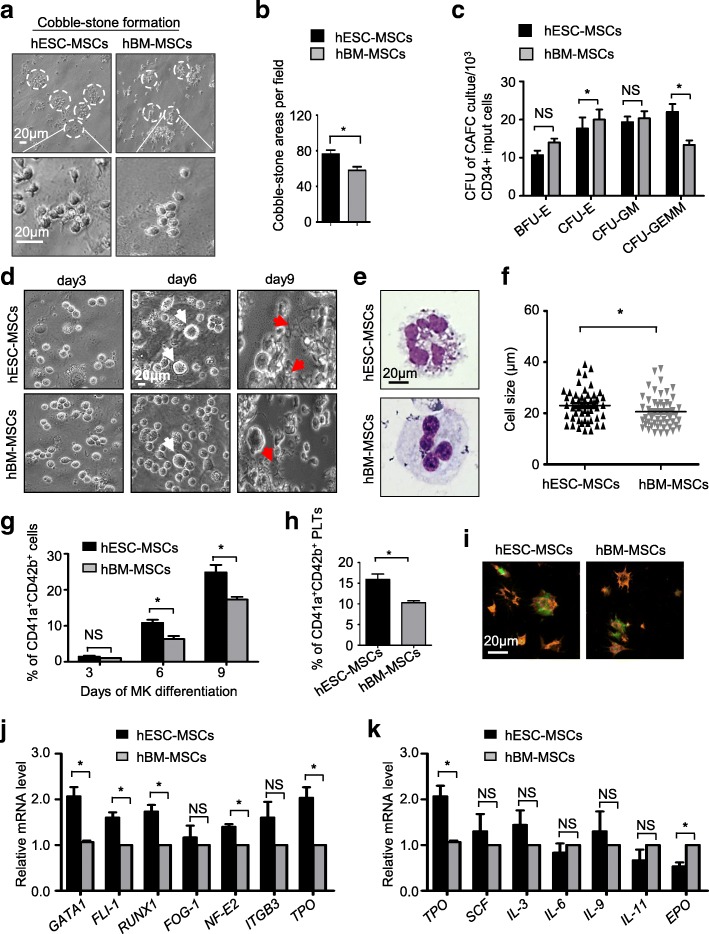
The hESC-MSCs show comparable hematologic-supporting potential as hBM-MSCs in vitro. **a** Phase contrast images of cobblestone areas formed by UCB-CD34^+^ cells co-cultured with hESC-MSCs or hBM-MSCs in DMEM/F-12 media containing 10% FBS and 10% horse serum for 2 weeks. Scale bar = 20 μm. **b** Statistical analysis of cobblestone areas per field between the hESC-MSC and hBM-MSC groups. Data are shown as mean ± SEM (*N* = 3). **c** Statistical analysis of colony-forming units (BFU-E, CFU-E, CFU-GM, CFU-GEMM) by UCB-CD34^+^ cell-derived cobblestone area-forming cells (CAFCs). Data are shown as mean ± SEM (*n* = 3). *P < 0.05; NS, not significant. **d** Representative cell morphology at day 3, day 6, or day 9 of megakaryocytic differentiation from UCB-CD34^+^ cells co-cultured with hESC-MSCs or hBM-MSCs in megakaryocytic differentiation media. Scale bar = 20 μm. Large cells are pointed by white arrows, and proplatelets are pointed by red arrows. **e** Representative megakaryocyte derived from UCB-CD34^+^ cells co-cultured with hESC-MSCs or hBM-MSCs at day 9, identified by Wright’s Giemsa staining. Scale bar = 20 μm. **f** Distribution of sizes of megakaryocytes at day 6 in **d** as measured with microscopy. Data are shown as mean ± SEM (*n* = 50). *P < 0.05. **g** Flow cytometry (FCM) analysis for the percentage of CD41a^+^CD42b^+^ megakaryocytes in the hESC-MSC and hBM-MSC groups at day 3, day 6, or day 9 of megakaryocytic differentiation, respectively. Data are shown as mean ± SEM (*n* = 3). *P < 0.05. **h** Flow cytometry (FCM) analysis for the percentage of CD41a^+^CD42b^+^ platelets in the hESC-MSC and hBM-MSC groups at day 9 of megakaryocytic differentiation. Data are shown as mean ± SEM (*n* = 3). *P < 0.05. **i** Aggregates of a mixture of Calcium-AM (red)-labeled blood or cultured platelets and blood platelets. In red, β-tubulin staining of both populations. Scale bar = 20 μm. **j** qRT-PCR analysis of the hematopoietic or megakaryocytic markers (*GATA1*, *FLI-1*, *RUNX1*, *FOG-1*, *NF-E2*, *ITGB3*, *TPO*) in CD34^+^ HSC-derived cells co-cultured with hESC-MSCs and hBM-MSCs at day 9. Data are shown as mean ± SEM (*n* = 3). *P < 0.05; NS, not significant. All values are normalized to the hBM-MSC group (= 1). **k** qRT-PCR analysis of the cytokines associated with hematopoietic or megakaryocytic differentiation (*TPO*, *SCF*, *IL-3*, *IL-6*, *IL-9*, *IL-11*, *EPO*) in hESC-MSCs and hBM-MSCs. Data are shown as mean ± SEM (*n* = 3). *P < 0.05; NS, not significant. All values are normalized to the hBM-MSC group (= 1)

Hematopoietic-supporting ability is a unique property of adult MSCs, yet it is largely unknown for hESC- or hiPSC-derived MSCs. To systematically explore the potential, we firstly conducted cobblestone formation assay as previously reported. After 4 weeks’ co-culture, more cobblestone areas with more than 5 cells were observed in the hESC-MSC group than those in the hBM-MSC group (hESC-MSCs vs hBM-MSCs, 76.00 ± 4.73 vs 58.00 ± 4.04, *P* = 0.0444) (Fig. 4a, b). Then, by practicing colony-forming unit (CFU) assay, we found the co-cultured cobblestone-forming cells could form different types of colonies, including BFU-E, CFU-E, CFU-GM, and CFU-GEMM, indicating the maintenance of the property of CD34^+^ hematopoietic progenitor cells (HPCs) (Additional file 4: Figure S4a). In details, when compared to the hBM-MSC group, more CFU-GEMM colonies and less CFU-E colonies were formed in the hESC-MSC group (Fig. 4c). In general, these data showed evidence that the derived hESC-MSCs were sufficient to support self-renewal of HPCs as hBM-MSCs.

Next, we were curious about whether the derived cells could also facilitate hematopoietic differentiation. For the purpose, we took advantage of our reported megakaryocytic differentiation procedure and co-cultured the purified umbilical cord blood CD34^+^ cells (UCB-CD34^+^) with hESC-MSCs or hBM-MSCs for 9 days. Compared to the hBM-MSC group, a significantly greater amount of cells with larger sizes, identified as megakaryocytes, began to arise in the hESC-MSC group at day 6 (Fig. 4d–f). In keeping with the cell size analysis, we observed more megakaryocytes and significantly higher percentages of the CD41a^+^CD42b^+^ MKs in the hESC-MSC group at day 6 (hESC-MSCs vs hBM-MSCs, 10.81% ± 0.87% vs 6.34% ± 0.82%, *P* = 0.020) and day 9 (hESC-MSCs vs hBM-MSCs, 24.87% ± 2.03% vs 17.32% ± 0.72%, *P* = 0.0247) (Fig. 4g, Additional file 4: Figure S4b). However, the total number of UCB-CD34^+^-derived cells showed no significant difference between the two groups (Additional file 4: Figure S4c).

Subsequently, we further tested whether the derived megakaryocytes were able to produce functional platelets. As expected, more filamentous proplatelets, followed by the release of genuine platelets, were observed in the hESC-MSC group (Fig. 4d). Quantitative analysis further confirmed the higher efficiency of generating CD41a^+^CD42b^+^ platelets at day 9 of megakaryocytic differentiation (hESC-MSCs vs hBM-MSCs, 15.91% ± 1.31% vs 10.30% ± 0.43%, *P* = 0.015) (Fig. 4h, Additional file 4: Figure S4d-S4e). Structure assay by immunofluorescence staining further indicated the typical discoid shapes and smooth contours of the platelets (Additional file 4: Figure S4f). Finally, to determine whether the derived platelets were functional, we practiced aggregation test of platelets. Interestingly, the derived platelets by co-culturing with hESC-MSCs or hBM-MSCs could interact with platelets segregated from peripheral blood and formed aggregates under thrombin-stimulating conditions (Fig. 4i). Next, we measured the expression of *TPO*, a key factor for megakaryopoiesis, and MK-associated genes such as GATA1, FLI-1, RUNX1, and NF-E2 in co-cultured UCB-CD34^+^ HSC-derived cells at day 9. Compared with the hBM-MSC group, higher levels of the abovementioned genes were expressed in the hESC-MSC group (Fig. 4j). Meanwhile, in accordance with our previous report [15], we also observed increased expression of TPO in hESC-MSCs (Fig. 4k). Taken together, these analyses demonstrated the derived hESC-MSCs were functional and sufficient for hematopoiesis in vitro.

### The hESC-MSCs could significantly improve the function of ischemic hind limbs in vivo

**Fig. 5:**
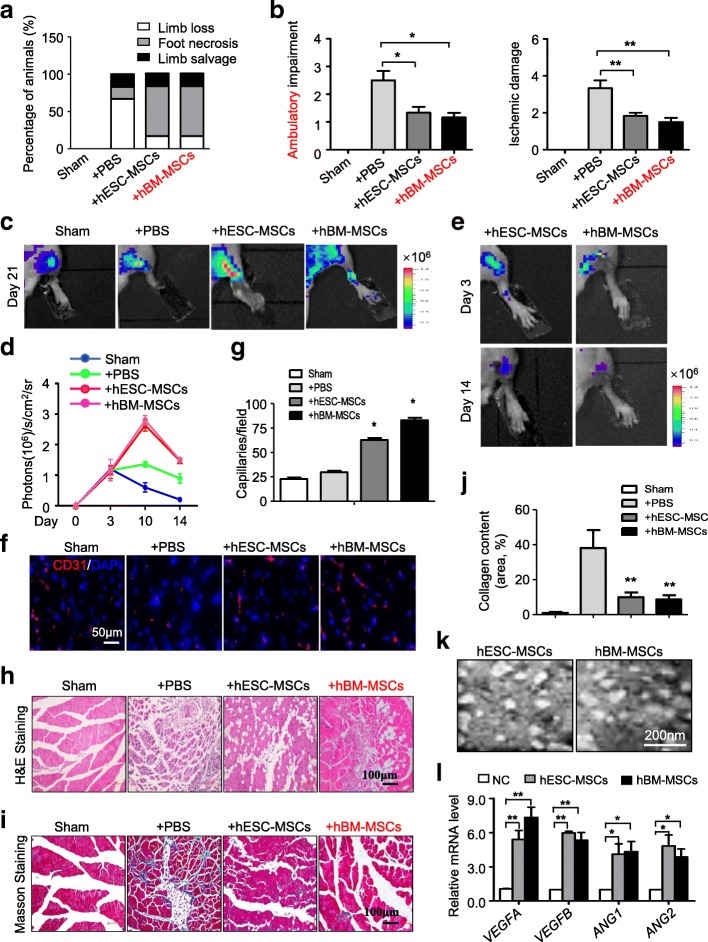
The hESC-MSCs could significantly improve the function of ischemic hind limbs in vivo. **a** Percentage distributions of limb loss, foot necrosis, and limb salvage were analyzed by Fisher’s exact test. **b** Semi-quantitative clinical assessments of ambulatory impairment (left) and ischemic damage (right) of ischemia hind limbs among the Ctr, +PBS, +hESC-MSC, and hBM-MSC groups. Data are shown as mean ± SEM (*n* = 6). *P < 0.05; **P < 0.01. **c** In vivo dynamically monitoring the status of angiogenesis after transplantation of hESC-MSCs or hBM-MSCs through tracking Vegfr2-luc expression by BMI in hind limb ischemic models at day 21. **d** Dynamic tendencies of angiogenesis in hind limbs based on Fluc signals among the indicated groups. **e** In vivo monitoring the retention of hESC-MSCs or hBM-MSCs by BLI analysis. **f** Representative images of hind limb muscle sections stained for CD31 (red) at day 14. DAPI was used for nucleus staining. **g** Quantification of capillary density of ischemic hind limbs in the indicated groups. Data are shown as mean ± SEM (*N* = 3). *P < 0.05; ^#^*P* < 0.05. **h** Histopathologic analysis of muscle tissues of mice (Ctr, +PBS, +hESC-MSCs) at day 28 by hematoxylin and eosin (H&E) staining (scale bar = 100 μm). **i** Histopathologic analysis of muscle tissues of mice (Ctr, +PBS, +hESC-MSCs) at day 28 by Masson staining (scale bar = 100 μm). **j** Quantitative analysis of collagen content in fibrotic area with Masson staining (Ctr, +PBS, +hESC-MSCs). Data are shown as mean ± SEM (*n* = 6). **P < 0.01. **k** Typical morphology of hESC-MSCs or hBM-MSCs secreted exosome under transmission electron microscopy. Scale bar, 200 nm. **l** qRT-PCR analysis of the cytokines associated with angiogenesis (*VEGFA*, *VEGFB*, *ANG1*, *ANG2*) in HUVECs after culturing with hESC-MSCs and hBM-MSCs for 2 days. Data are shown as mean ± SEM (*n* = 3). *P < 0.05; **P < 0.01. All values are normalized to the NC group (= 1)


Previously, we have demonstrated that adult tissue-derived MSCs could alleviate the severity of hind limb ischemia [27, 29]. Thus, we took advantage of this model to explore whether hESC-MSC transplantation could also have similar therapeutic potential in vivo. As we previously reported, we classified mice into the three outcomes including limb loss, foot necrosis, and limb salvage. Compared to the other three groups, intramuscular injection of hESC-MSCs reduced the severity of hind limb ischemia to some extent (Additional file 5: Figure S5a). In coincidence, statistical analysis of three outcomes by Fisher’s exact test further showed a significant decrease of the amputation rate in hESC-MSC and hBM-MSC groups (Fig. 5a). Hind limb function detection at day 8 showed that both hESC-MSC and hBM-MSC transplantation could help restore hind limb function (Additional file 5: Figure S5b, Additional files 6, 7, 8 and 9: Videos for hind limb function detection). Meanwhile, in the assessment of the ischemic status based on the ambulatory impairment, we found hESC-MSCs assuaged the ambulatory impairment (+PBS vs +hESC-MSCs, 2.50 ± 0.34 vs 1.33 ± 0.22, *P* = 0.00157) and ischemic damage (+PBS vs +hESC-MSCs, 3.33 ± 0.42 vs 1.83 ± 0.17, *P* = 0.0079) when compared with the +PBS group, respectively (Fig. 5b).

Additional file 6: Additional information. Videos for hind limb function detection of mice in the sham group at day 8 of the model. (MOV 17949 kb)



Additional file 7: Additional information. Videos for hind limb function detection of mice in the +PBS group at day 8 of the model. (MOV 13848 kb)



Additional file 8: Additional information. Videos for hind limb function detection of mice in the +hESC-MSC group at day 8 of the model. (MOV 13351 kb)



Additional file 9: Additional information. Videos for hind limb function detection of mice in the +hBM-MSC group at day 8 of the model. (MOV 19491 kb)


To investigate the proangiogenic effect of hESC-MSCs and hBM-MSCs in vivo, we took advantage of the transgenic Vegfr2-luc mice as we previously reported [30]. Therefore, we could detect the angiogenesis and conduct hemodynamic monitoring via imaging. Compared with the +PBS group, we could clearly notice stronger signals in the +hESC-MSC and +hBM-MSC groups (Fig. 5c, d). Meanwhile, we utilized the abovementioned system to detect the retention of MSCs in vivo. With the aid of coelenterazine (5 mg/kg) and IVIS Lumina Imaging System, the dynamic distributions of Gluc-labeled hESC-MSCs and hBM-MSCs were monitored in the ischemic hind limbs (Fig. 5e). Additionally, immunostaining showed that anti-human Ki-67^+^ or α-SMA^+^ cells could be detected in both hESC-MSC- and hBM-MSC-treated tissues (Additional file 5: Figure S5c-S5d), which indicated the superiority of hESC-MSCs in proliferation and retention in vivo, respectively.

Furthermore, at day 14 of the disease model, the mice were decapitated and the ischemic muscles in the hind limb were fixed for assessing the beneficial effect of hESC-MSC transplantation. The CD31 immunostaining showed that microvascular density in ischemia hind limbs was significantly enhanced by hESC-MSC or hBM-MSC transplantation (Fig. 5f, g), which was consistent with the above BLI data. Aided with hematoxylin and eosin staining (H&E staining), we noticed a significant reduction of the degeneration and apoptosis of the fibers together with less inflammatory cells in the hESC-MSC group (Fig. 5h). In addition, at day 28, the corrective effect of hESC-MSC transplantation on fibrosis were confirmed with Masson staining and the quantitative analysis of collagen content in fibrotic area (+PBS vs +hESC-MSCs, 39.83% ± 9.67% vs 8.33% ± 1.91%, *P* = 0.0096) (Fig. 5i, j, Additional file 5: Figure S5e). To explore the underlying mechanism, we firstly enriched the supernatant after culturing hESC-MSCs or hBM-MSCs for 2 days and detected the expression level of angiogenesis-associated proteins such as VEGF, bFGF, and SDF-1α. Quantitative analysis showed that hESC-MSCs secreted more bFGF but less SDF-1α than hBM-MSCs (Additional file 5: Figure S5f). Then, the exosomes secreted by hESC-MSCs or hBM-MSCs were collected and confirmed by electron microscopy and protein detection (Fig. 5k, Additional file 5: Figure S5 g). To dissect the proangiogenic potential of hESCs, we carried out tube formation assay of HUVECs with exosome addition as we recently reported. Consistently, we found that both hESC-MSC- and hBM-MSC-secreted exosomes could increase expression of proangiogenic genes such as *VEGFA*, *VEGFB*, *ANG1*, and *ANG2* in the endothelial cells (Fig. 5l), which was consistent with the in vivo data. Taken together, these data indicated the therapeutic effects of hESC-MSCs on hind limb ischemia by extensive protection of the endangered muscle cells and fibers.

## Discussion

In this study, we established a rapid and high-efficiency procedure for MSC generation from hESCs with the aid of JNKi and DAC in 2 weeks. Furthermore, we found the derived cells satisfied the criteria of adult MSCs, including spindle-shaped morphology, surface antigen expression, multi-lineage differentiation capacity, and normal chromosome karyotype, but with superiority in long-term proliferation ability. Functionally, compared with adult MSCs (hBM-MSCs), hESC-MSCs had preferable hematopoietic-supporting capacity demonstrated by the properties of benefiting HPC long-term maintenance and facilitating megakaryocytic differentiation. Conclusively, the therapeutics of hESC-MSCs revealed beneficial effects on structural and functional recovery of ischemic muscles in the hind limb ischemia model.

Previously, we and other investigators have demonstrated the unique properties of adult tissue-derived MSCs in fundamental research and clinical application [6, 8, 12, 13, 19]. However, those cell sources have a number of limitations as well, such as limited long-term in vitro proliferative capacity, donor-specific variability in quality, onset of replicative senescence, and decreased therapeutic potency after continuous in vitro passages [13]. Thus, there is an urgency to look for alternative inexhaustible and homogeneous sources of MSCs. For decades, numbers of procedures for high-efficiency generation of MSCs from hESCs or hiPSCs were reported, including the co-culture, monolayer, and embryoid body (EB) model [12, 23–25]. Although a certain number of MSCs were obtained, the subsistent shortcomings of most current procedures hampered large-scale production and their clinical application [13]. Herein, we have established a convenient two-step method aided with JNKi and DAC for considerable quantities of MSC generation within 2 weeks without the requirement for laborious manipulations, such as cell sorting, handpicking, or serial passages. Most importantly, in contrast to the current strategies with time-consuming or low-efficient disadvantages, it is more feasible and convenient to uncover the developmental process and molecular signatures of MSCs with this programming approach [13, 31]. Although the exact underlying mechanism during mesenchymal differentiation is largely unknown except for NF-κB signal, TGF-β signal, MSX2, and EZH2 [13, 21, 22, 24], our findings indicate JAK/STAT signaling pathway and epigenetic modification may play an important role in MSC generation.

MSCs are promising sources for regenerative medicine and cell-based therapies due to their unique characteristics in supporting hematopoiesis and immunomodulatory properties [1, 32]. Despite the encouraging advances of adult MSCs in hematopoietic reconstruction, the function of hESC-MSCs is still largely unknown. In this study, we investigated the effect of hESC-MSCs in modulating HPC functions both under the maintenance condition and under megakaryocytic differentiation condition. Interestingly, in keeping with adult MSCs (hBM-MSCs), hESC-MSCs were sufficient to support the proliferative HPCs to form cobblestone-like colonies or to differentiate into mature megakaryocytes with platelet release. These results indicate potential applications of hESC-MSCs in the long-term in vitro maintenance of HPCs and for large-scale generation of functional platelets.

In addition, we and other investigators found that the hESCs- or hiPSC-derived MSCs show therapeutic effect on series of animal models of human diseases, including acute colitis, multiple sclerosis, and experimental inflammatory bowel disease [13, 18, 20]. In this study, we found hESC-MSC transplantation increased myogenesis and partially restored limb function, which provided new evidences for the therapeutic effect of hESC-MSCs on hind limb ischemia. Our results were in parallel with the previous report that iPSC-MSC transplantation could attenuate tissue ischemia by increasing myogenesis and neovascularization [26]. Hence, in consideration of the shortages and deficiencies of adult tissue-derived MSCs, hESC-MSCs will have greater potential in regenerative medicine especially for their superiorities in robust proliferation and long-term viability. Additionally, there is also an urgency to systemically illuminate the biological functions and molecular signatures of hESC-MSCs [13, 21]. For instance, in this study, we found hESC-MSCs could facilitate hematopoiesis and alleviate hind limb ischemia simultaneously. We may speculate that hESC-MSCs express higher level of exosomes or cytokines such as TPO and VEGF, yet the link and exact functional mechanisms are largely unknown.

Above all, due to the absence of high-efficiency procedure for hESC-MSC generation, very limited studies focused on dissecting the biological process and molecular mechanism of mesenchymal differentiation from hPSCs [13, 21, 22]. Herein, we established a two-step strategy for rapid and robust induction of MSC with the aid of two chemical compounds. Thus, it will be feasible and intriguing to further explore the biological signatures and mechanisms underlying the mesenchymal differentiation process in the future.

## Conclusions

Overall, in this study, we have established a rapid and high-efficiency procedure for homologous MSC generation from hESCs. The derived hESC-MSCs were similar to adult MSCs but with superiorities in supporting hematopoiesis and megakaryopoiesis. Above all, the programming strategy could be of enormous potential for regenerative medicine such as hind limb ischemia treatment.

## Additional files

Additional file 1: **Figure S1.** Identification of hESC-MPCs and hBM-MSCs by flow cytometry. (PDF 155 kb)

Additional file 2: **Figure S2.** Identification of hESC-MSCs by flow cytometry, qRT-PCR, western blotting, and immunostaining analysis. (PDF 199 kb)

Additional file 3: **Figure S3.** In vivo tissue and in vitro multi-lineage differentiation potential of hESC-MSCs. (PDF 348 kb)

Additional file 4: **Figure S4.** Identification of hematopoietic-supporting effect of hESC-MSCs. (PDF 232 kb)

Additional file 5: **Figure S5.** Identification of the therapeutic effect of hESC-MSCs on alleviating hind limb ischemia by immunostaining, qRT-PCR, western blotting, and ELISA analyses. (PDF 443 kb)

Additional file 10: Additional information. The details accompanied with the main manuscript including additional figure legends, additional experimental procedures, additional tables, and additional references were listed. (DOCX 38 kb)

## Data Availability

All data generated or analyzed during this study are included in this published article and its supplementary information files. Meanwhile, the datasets used and analyzed during the current study are also available from the corresponding author on reasonable request.

## Abbreviations

AD-MSCs: Adipose-derived MSCs
BFU-E: Burst-forming unit erythroid
CFU-E: Colony-forming unit erythrocyte
CFU-GEMM: Colony-forming unit granulocyte/erythroid/macrophage/megakaryocyte
CFU-GM: Colony-forming unit granulocyte/macrophage
ELISA: Enzyme-linked immunosorbent assay
H&E: Hematoxylin and eosin
MKs: Megakaryocytes
MSCs: Mesenchymal stem/stromal cells
Pd: Population doubling
PGE1: Prostaglandin E1
