# Supplementary figures and images for: JNKi- and DAC-programmed mesenchymal stem/stromal cells from hESCs facilitate hematopoiesis and alleviate hind limb ischemia
Source: Stem Cell Res Ther. 2019 Jun 24;10:186. doi: 10.1186/s13287-019-1302-1 (PMC6591900; doi:10.1186/s13287-019-1302-1)

**a**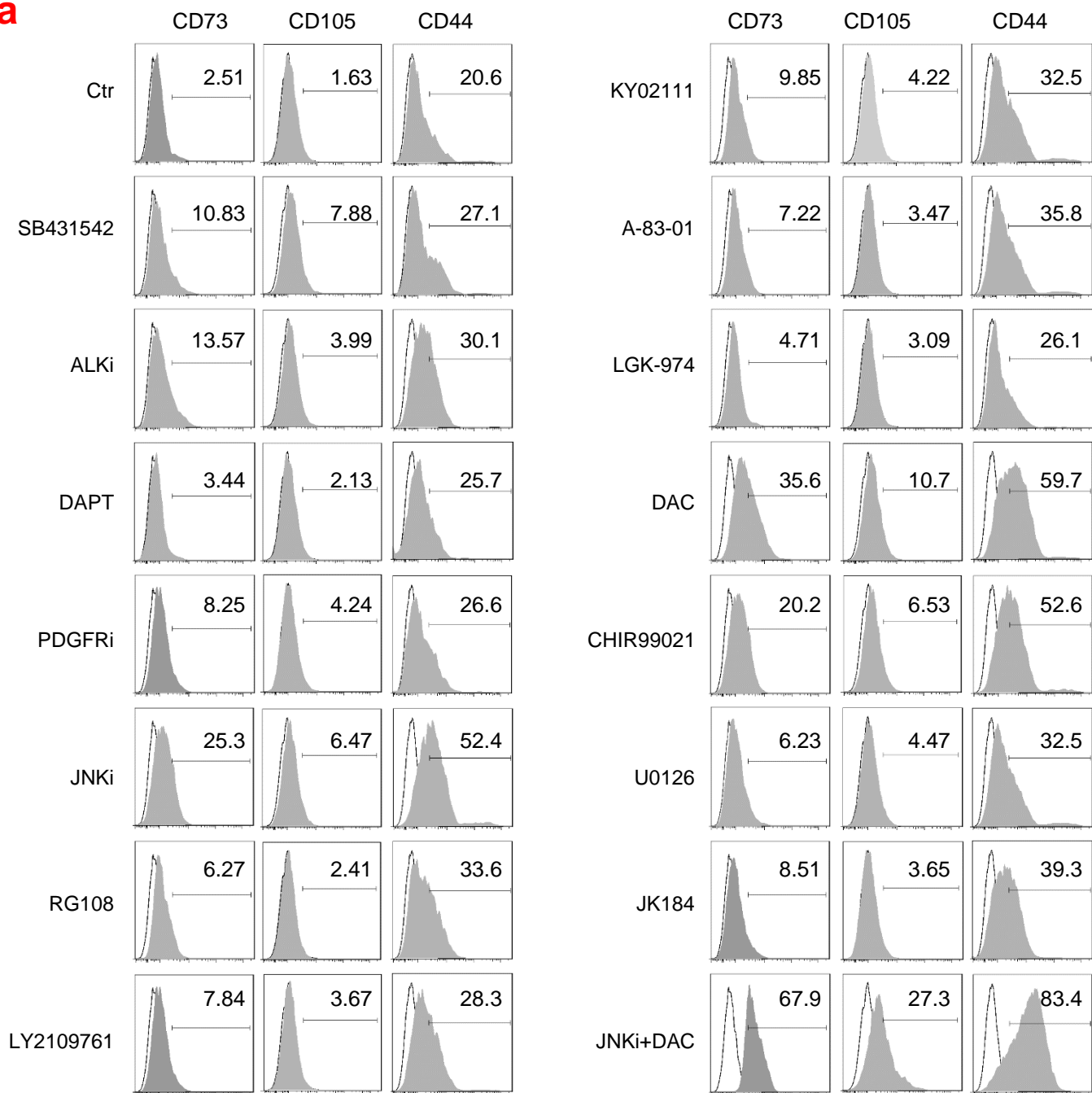**b**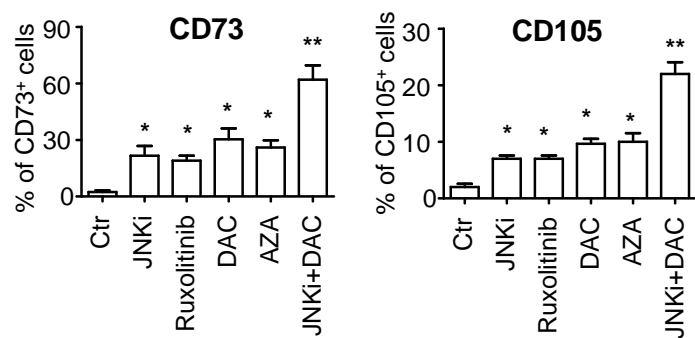**c**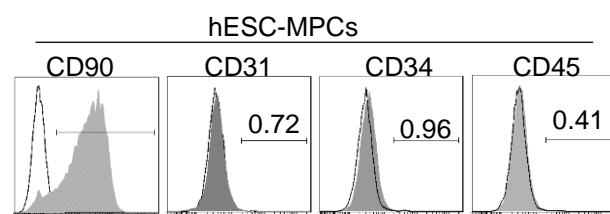

Supplement: Supplementary file 5 — Figure S1. Identification of hESC-MPCs and hBM-MSCs by flow cytometry. (PDF 155 kb) [file 13287_2019_1302_MOESM1_ESM.pdf]

**a**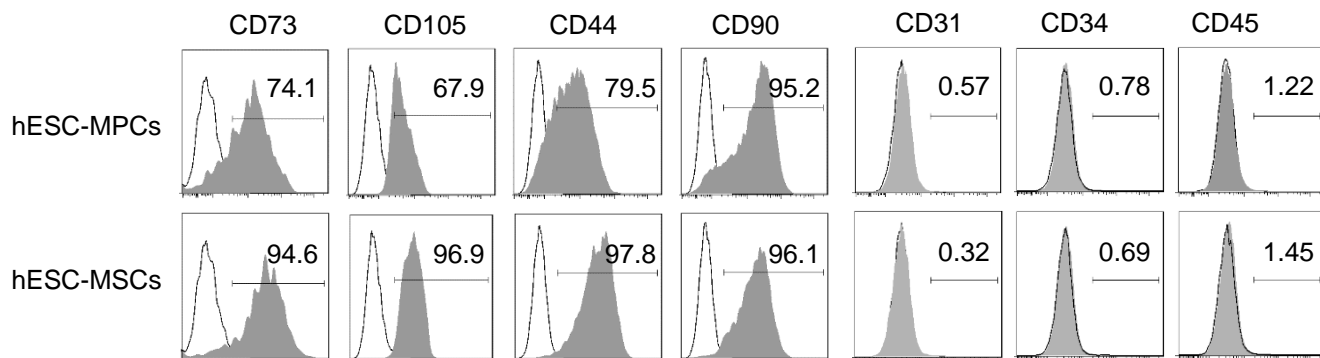**b**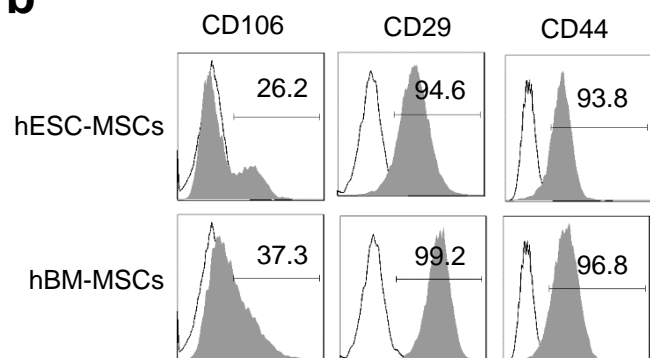**c**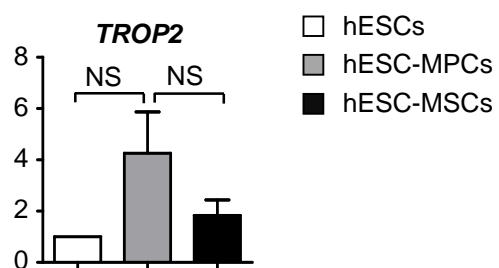**d**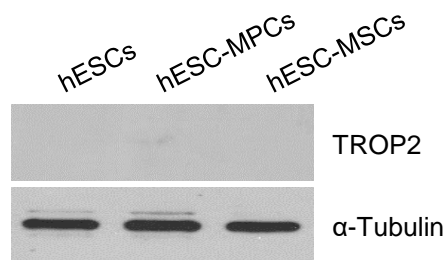**e**

□ hESCs    ■ hESC-MPCs    ■ hESC-MSCs

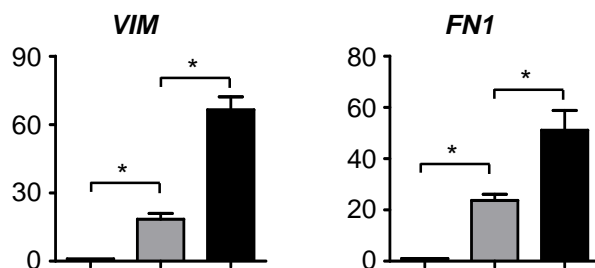**f**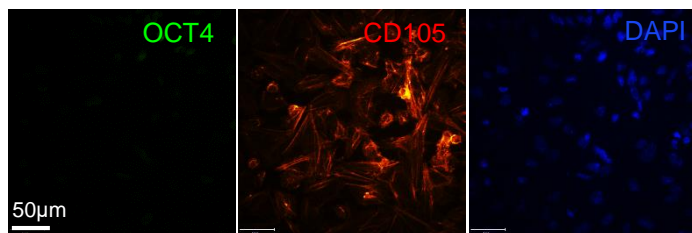

Supplement: Supplementary file 6 — Figure S2. Identification of hESC-MSCs by flow cytometry, qRT-PCR, western blotting, and immunostaining analysis. (PDF 199 kb) [file 13287_2019_1302_MOESM2_ESM.pdf]

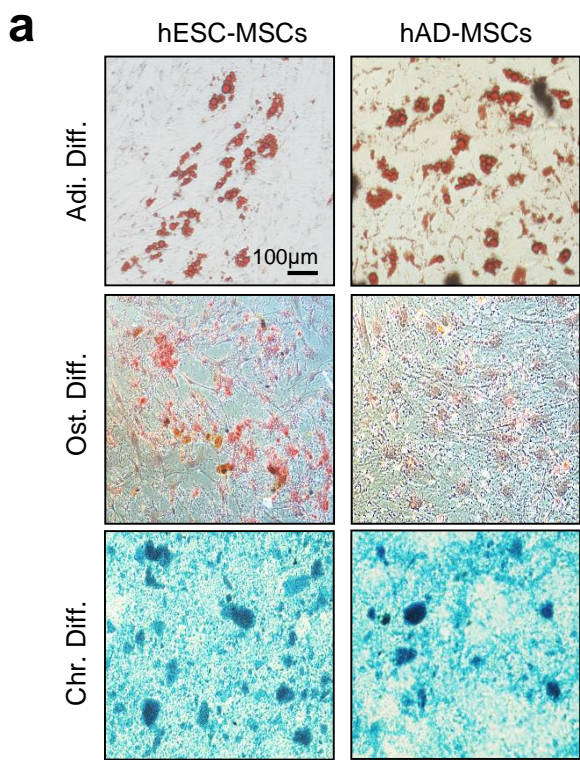

**b**

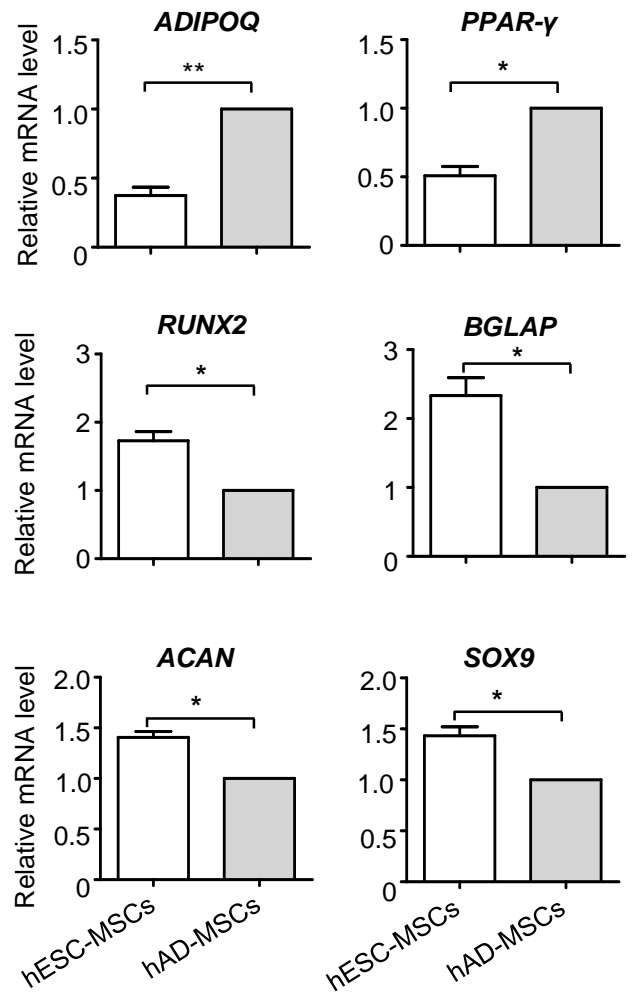

**c**

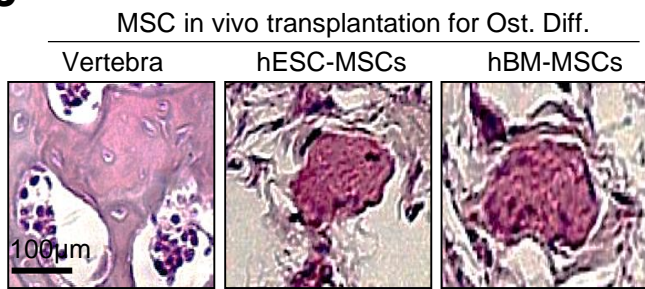

Supplement: Supplementary file 7 — Figure S3. In vivo tissue and in vitro multi-lineage differentiation potential of hESC-MSCs. (PDF 348 kb) [file 13287_2019_1302_MOESM3_ESM.pdf]

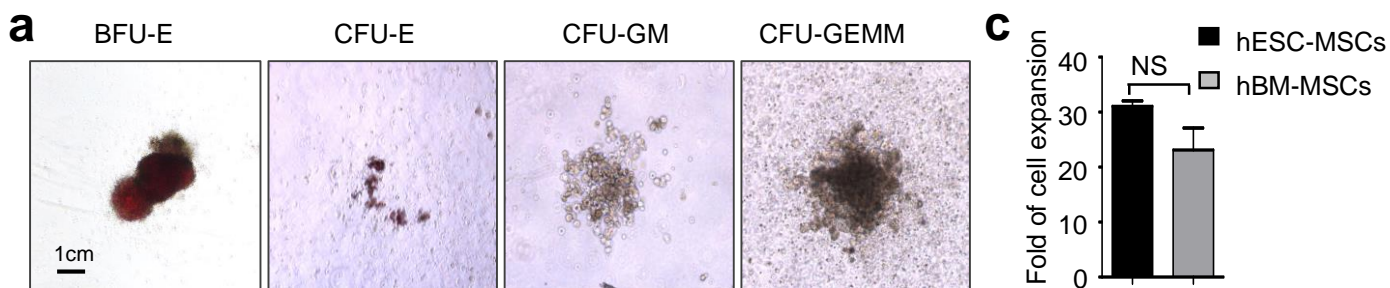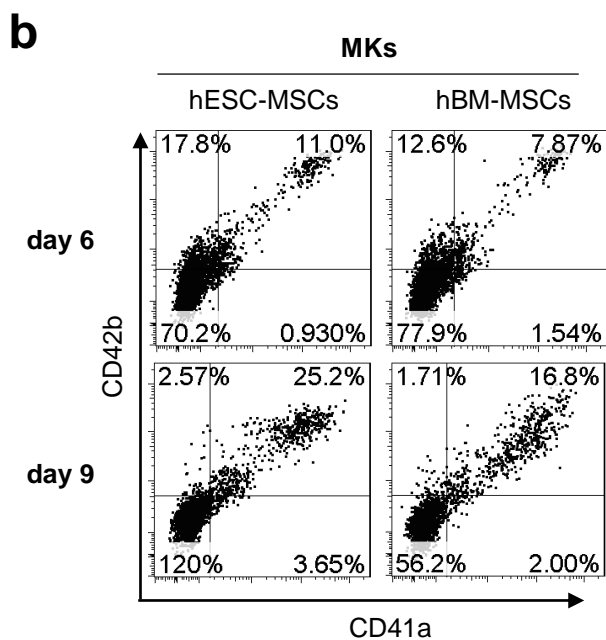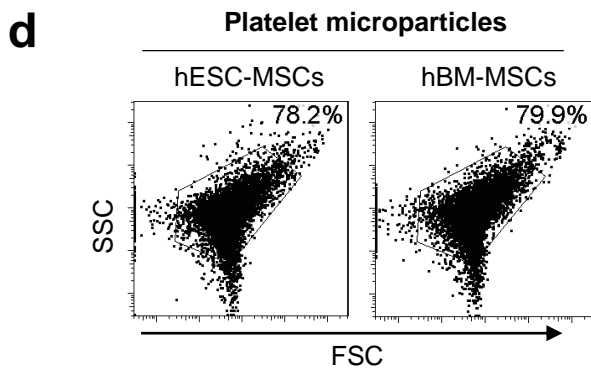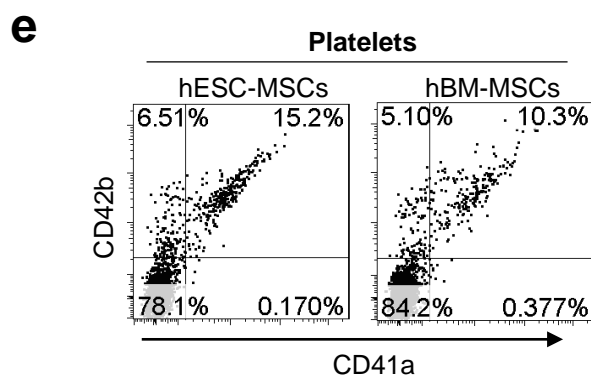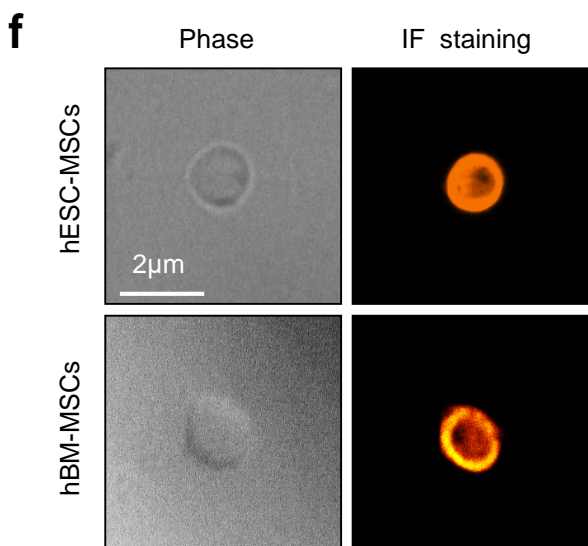

Supplement: Supplementary file 8 — Figure S4. Identification of hematopoietic-supporting effect of hESC-MSCs. (PDF 232 kb) [file 13287_2019_1302_MOESM4_ESM.pdf]

**a**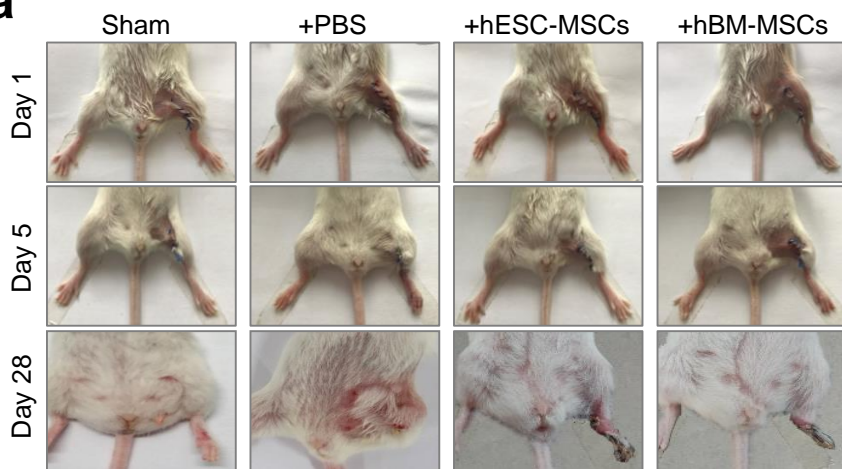**c**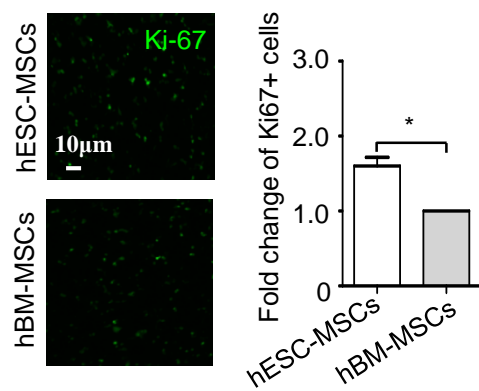**b**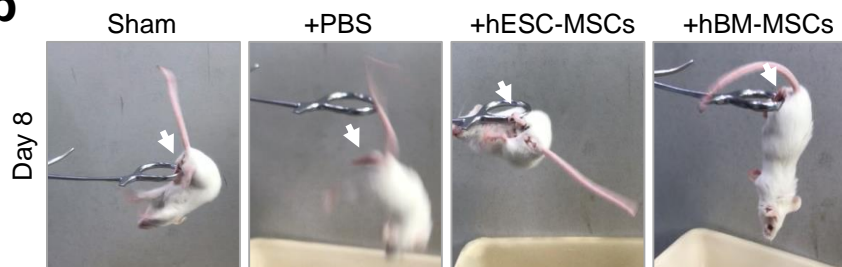**d**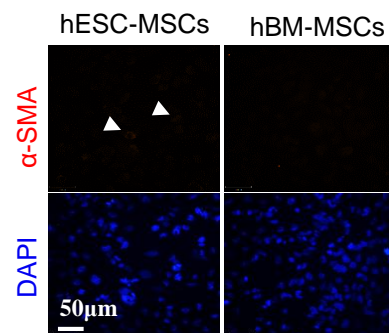**e**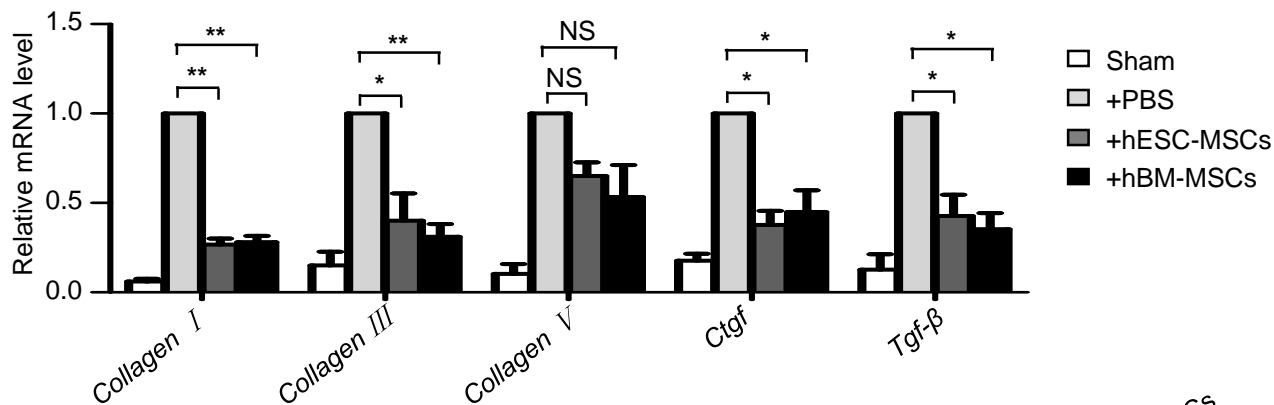**f**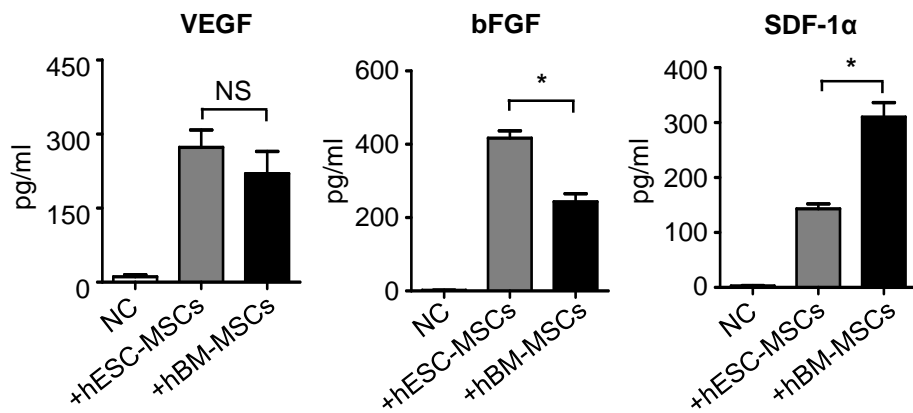**g**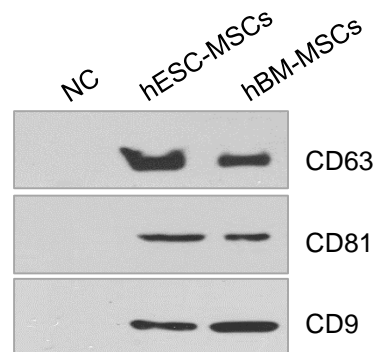

Supplement: Supplementary file 9 — Figure S5. Identification of the therapeutic effect of hESC-MSCs on alleviating hind limb ischemia by immunostaining, qRT-PCR, western blotting, and ELISA analyses. (PDF 443 kb) [file 13287_2019_1302_MOESM5_ESM.pdf]
