# Supplementary material for: JNKi- and DAC-programmed mesenchymal stem/stromal cells from hESCs facilitate hematopoiesis and alleviate hind limb ischemia
Source: Stem Cell Res Ther. 2019 Jun 24;10:186. doi: 10.1186/s13287-019-1302-1 (PMC6591900; doi:10.1186/s13287-019-1302-1)
Supplement: Supplementary file 10 — Additional information. The details accompanied with the main manuscript including additional figure legends, additional experimental procedures, additional tables, and additional references were listed. (DOCX 38 kb) [file 13287_2019_1302_MOESM10_ESM.docx]

**Additional Information**

**JNKi and DAC Programmed Mesenchymal Stem/Stromal Cells from hESCs Facilitate Hematopoiesis and Alleviate Hind Limb Ischemia**

Yimeng Wei^1^**^§^**, Huixing Hou^2,3^**^§^**, Leisheng Zhang^1,3-7^**^*^**, Nianhuan Zhao^2,3^, Chengwen Li^1^, Ying Liu^1^, Wenxia Zhang^1^, Zongjin Li^3,4^, Dengke Liu^5^, Zhibo Han^1,7^, Lei Zhang^1^, Baoquan Song^8*^, Ying Chi^1*^, Zhongchao Han^1,6,7*^

**Additional Figure Legends for Addition Fig S1-S5;**

**Additional Experimental Procedures;**

**Additional Tables 1-3;**

**Additional References.**

**Figure S1. Relative to Figure 1. Identification of hESC-MPCs and hBM-MSCs**

(a) Flow cytometry (FCM) analysis of MSC markers (CD73, CD105, CD44) of hESCs-derived cells cultured in 5% FBS/DMEM/F12 ± 10nM chemical compounds for 7 days. (b) Flow cytometry (FCM) analysis of MSC marker of hESCs-derived cells cultured in 5% FBS/DMEM/F12 ± 10nM chemical compounds for 7 days (mean ± SEM, N = 3). *p < 0.05;**p < 0.01. (c) Flow cytometry (FCM) analysis of MSC markers (CD90), endothelial marker (CD31), hematopoietic markers (CD34, CD45) of hESCs-MPCs cultured in 5% FBS/DMEM/F12 + 10nM JNKi and DAC for 7 days.

**Figure S2. Relative to Figure 2. Identification of hESC-MSCs**

(a) Flow cytometry (FCM) analysis of MSC markers (CD73, CD105, CD44, CD90), endothelial marker (CD31), hematopoietic markers (CD34, CD45) of hESCs-MPCs and hESC-MSCs. (b) Flow cytometry (FCM) analysis of MSC markers (CD106, CD29, CD44) of hESCs-MSCs and hBM-MSCs. (c) qRT-PCR analysis of germ layer associated genes (*TROP2*) in undifferentiated hESCs, hESC-MPCs and hESC-MSCs. Data are shown as mean ± SEM (n=3). NS, not significant. (d) Western-blotting analysis of germ layer associated genes (*TROP2*) in undifferentiated hESCs, hESC-MPCs and hESC-MSCs. α-Tubulin was used as a loading control. (e) qRT-PCR analysis of MSC-associated genes (*VIM, FN1*) in undifferentiated hESCs, hESC-MPCs and hESC-MSCs. Data are shown as mean ± SEM (n=3). *, *P*<0.05; NS, not significant. All values are normalized to the hESCs group (=1). (f) Representative immunofluorescence images displays the expression of OCT4 (in green), CD105 (in red) in hESCs (scale bar=50μm). The nuclei (in blue) was labeled with DAPI.

**Figure S3. Relative to Figure 3. *In vivo* tissue and *in vitro* multi-lineage differentiation potential of hESC-MSCs**

(a) Multi-lineage differentiation potential of hESC-MSCs and hAD-MSCs was identified by Oil red O staining, Alizarin red staining or Alcian blue staining. (b) qRT-PCR analysis of the adipogenic (*ADIPOQ*, *PPAR-γ*), osteogenic (*RUNX2*, *BGLAP*) or chondrogenic (*ACAN*, *SOX9*) markers in hESC-MSCs and hBM-MSCs. Data are shown as mean ± SEM (n=3). *, *P*<0.05; **, *P*<0.01; NS, not significant. All values are normalized to the hAD-MSCs group (=1). (c) The s.c. in-vivo transplantation assay using immunodeficient mice to evaluate the bone tissue differentiation potential of hESC-MSCs and hAD-MSCs. Scale bar, 100 μm.

**Figure S4. Relative to Figure 4.** **Identification of Hematopoietic-supporting effect of hESC-MSCs**

(a) Hematopoietic colony-forming potential of UCB-CD34^+^ cell-derived cobblestone area-forming cells (CAFCs) detached from the co-cultured groups (hESC-MSCs, hBM-MSCs). Representative morphologies of BFU-E, CFU-E, CFU-GM and CFU-GEMM are shown. Scale bar=1cm. (b) Fold change of UCB-CD34^+^ cell-derived cells at day6 of megakaryocytic differentiation in the hESC-MSCs or hBM-MSCs group to those at day0. Data are shown as mean ± SEM (n = 3). NS, not significant. (c) Flow cytometer (FCM) analysis for the percentage of CD41a^+^CD42b^+^ megakaryocytes in the hESC-MSCs and hBM-MSCs groups at day6 or day9 of megakaryocytic differentiation, respectively. (d) Representative flow cytometer analysis for the percentage of platelet microparticles at day9 of megakaryocytic differentiation from UCB-CD34^+^ HSC cocultured with hESC-MSCs or hBM-MSCs. (e) Flow cytometer (FCM) analysis for the percentage of CD41a^+^CD42b^+^ platelets in the hESC-MSCs and hBM-MSCs groups at day9 of megakaryocytic differentiation. (f) Phase contrast images (left panel) and fluorescence images (right panel) of co-cultured platelets in hESC-MSCs or hBM-MSCs group. Platelets are stained for β-tubulin (microtubule cytoskeleton, orange). Scale bar=2μm.

**Figure S5. Relative to Figure 5. Identification of the therapeutic effect of hESC-MSCs on alleviating hind limb ischemia**

(a) Representative photographs of hind limbs of ischemia model mice after injection of 1×PBS (+PBS), hESC-MSCs (+hESC-MSCs) or hBM-MSCs (+hBM-MSCs). Untreated mice (Sham) were served as control. (b) Representative photographs of hind limbs of ischemia model mice during functional detection. (c-d) Representative images of hind limb muscle sections stained for Ki-67 at day 14 (c) and α-SMA (Red) at day28 (d). DAPI was used for nucleus staining. (e) qRT-PCR analysis of *Collagen I*, *Collagen III*, *Collagen IV*, *Ctgf* and *Tgf-β* in muscles of hind limbs of mice. Data are shown as mean ± SEM (n=3). *, *P*<0.05; **, *P*<0.01; NS, not significant. All values are normalized to the +PBS group (=1). (f) Quantitative analysis of the expression level of angiogenesis-associated proteins (VEGF, bFGF, SDF-1α) after culturing for 2 days. The RPMI-1640 basic media was used as a negative control (NC). Data are shown as mean ± SEM (n = 3). *, *P*<0.05; NS, not significant. (g) Western blotting analysis of CD63, CD81, CD9 protein in hESC-MSCs or hBM-MSCs secreted exosome.

**Additional Experimental Procedures**

**Platelets Enrichment and Purification**

The platelets were enriched and purified as we previously reported[[1](#_ENREF_1)]. Briefly, the platelets in the supernatant (in the hESC-MSCs or hBM-MSCs group) were collected by centrifugation at 300×g for 10 minutes. Then, the platelets were resuspended in 2ml 1×CGS buffer with 1μM PGE1 (Sigma), and immediately spun at a BSA density [gradient](javascript:void(0);) [centrifugation](javascript:void(0);) of 800×g for 10 min. The upper layers containing the purified platelets were centrifuged for 10 min at 800×g. Finally, the purified platelets were resuspended again in 1×CGS buffer and maintained at room temperature for further assay.

**Immunofluorescence of Platelets**

Immunofluorescence of platelets were practiced as we previously reported[[1-3](#_ENREF_1)]. Briefly, platelets collected and purified from peripheral blood (PB) or derived by co-culture were plated onto 35mm confocal dishes (Corning) coated with poly-L-lysine (100μg/ml) at 37℃ for 1 hr. Then, these platelets were fixed, permeabilized and blocked as we previously did. For characterization assay, the platelets were labeled with mouse anti-β1-tubulin antibody and incubated with 594-conjugated donkey anti-mouse IgG (Invitrogen). Then, the fluorescent images of platelets (in the hESC-MSCs or hBM-MSCs group) were recorded under a confocal microscopy (LSM710).

**Additional Tables**

**Table S1. Primers used in this study.**

Real-time PCR primer sequences.

| Gene | Forward Primer | Reverse Primer |
| --- | --- | --- |
| *ACTIN* | CTCTTCCAGCCTTCCTTCCT | AGCACTGTGTGTTGGCGTACAG |
| *POU5F1* | CTTGAATCCCGAATGGAAAGGG | GTGTATATCCCAGGGTGATCCTC |
| *SOX2* | GCCGAGTGGAAACTTTTGTCG | GGCAGCGTGTACTTATCCTTCT |
| *NANOG* | TTTGTGGGCCTGAAGAAAACT | AGGGCTGTCCTGAATAAGCAG |
| *T* | CTGGGTACTCCCAATGGGG | GGTTGGAGAATTGTTCCGATGA |
| *GATA2* | ACTGACGGAGAGCATGAAGAT | CCGGCACATAGGAGGGGTA |
| *PAX6* | ATGTGTGAGTAAAATTCTGGGCA | GCTTACAACTTCTGGAGTCGCTA |
| *TROP2* | ACAACGATGGCCTCTACGAC | GTCCAGGTCTGAGTGGTTGAA |
| *NT5E* | GGGCGGAAGGTTCCTGTAG | GAGGAGCCATCCAGATAGACA |
| *ENG* | AGCCCCACAAGTCTTGCAG | GCTAGTGGTATATGTCACCTCGC |
| *VIM* | GAAGAGAACTTTGCCGTTGAAG | GAAGGTGACGAGCCATTT |
| *FN1* | TGACCCCTACACAGTTTCCCA | TGATTCAGACATTCGTTCCCAC |
| *ADIPOQ* | TGGTCCTAAGGGAGACATCG | TGGAATTTACCAGTGGAGCC |
| *PPAR-γ* | GCTGGCCTCCTTGATGAATA | TGTCTTCAATGGGCTTCACA |
| *RUNX2* | CTCACTACCACACCTACCTG | TCAATATGGTCGCCAAACAGATTC |
| *BGLAP* | GGCGCTACCTGTATCAATGG | TCAGCCAACTCGTCACAGTC |
| *ACAN* | CCCCTGCTATTTCATCGACCC | GACACACGGCTCCACTTGAT |
| *SOX9* | AATGGAGCAGCGAAATCAAC | CAGAGAGATTTAGCACACTGATC |
| *VEGFA* | TGTCTAATGCCCTGGAGCCT | GTCACATCTGCAAGTACGTTCG |
| *VEGFB* | GAGATGTCCCTGGAAGAACACA | GAGTGGGATGGGTGATGTCAG |
| *ANG1* | CGCCGAAGTCCAGAAAACAG | GGGAAGAGAAATCCGGTTCCA |
| *ANG2* | GCTCGAATACGATGACTCGGT | GTTTGCTCCGCTGTTTGGTT |

**Table S1 continued. Primers used in this study.**

Real-time PCR primer sequences.

| Gene | Forward Primer | Reverse Primer |
| --- | --- | --- |
| *TPO* | AACTGCAAGGCTAACGCTGT | GACATGGGAGTCACGAAGCA |
| *SCF* | AATCCTCTCGTCAAAACTGAAGG | CCATCTCGCTTATCCAACAATGA |
| *IL-3* | CAGACAACGCCCTTGAAGACA | GCCCTGTTGAATGCCTCCA |
| *IL-6* | ACTCACCTCTTCAGAACGAATTG | CCATCTTTGGAAGGTTCAGGTTG |
| *IL-9* | CTCTGTTTGGGCATTCCCTCT | GGGTATCTTGTTTGCATGGTGG |
| *IL-11* | CGAGCGGACCTACTGTCCTA | GCCCAGTCAAGTGTCAGGTG |
| *EPO* | GGAGGCCGAGAATATCACGAC | CCCTGCCAGACTTCTACGG |
| *GATA1* | CACTGAGCTTGCCACATCC | ATGGAGCCTCTGGGGATTA |
| *FLI-1* | GGCCTGAACAGTAGAGGCG | CACCGGAGACTCCCTGGAT |
| *RUNX1* | TCTTCACAAACCCACCGCAA | CTGCCGATGTCTTCGAGGTTC |
| *FOG-1* | CGTGCTTCGAGTGCGAGAT | CGCCTCTACTGTTCAGGCC |
| *NF-E2* | CGGCGCAGCGAATATGTAGA | CCGACGTTCATCCCGACTC |
| *ITGB3* | GTGACCTGAAGGAGAATCTGC | CCGGAGTGCAATCCTCTGG |

**Table S2. Antibodies used in this study.**

Antibodies for flow cytometry and immunofluorescence.

| Antibody | Cat. NO. | Source |
| --- | --- | --- |
| Anti-CD11b-PE | 557743 | BD Pharmigen |
| Anti-CD29-PE | 303003 | Biolegend |
| Anti-CD41a-APC | 555751 | BD Pharmigen |
| Anti-CD42b-PE | 555473 | BD Pharmigen |
| Anti-CD44-PE | 550989 | BD Pharmigen |
| Anti-CD73-Percp-cy5.5 | 46-0739-42 | eBioscience |
| Anti-CD90-FITC | 11-0909-42 | eBioscience |
| Anti-CD105-APC | 323208 | BioLegend |
| Anti-CD106-PE | 561679 | BD Pharmigen |
| Anti-CD31-PE | 560975 | BD Pharmigen |
| Anti-CD34-APC | 555824 | BD Pharmigen |
| Anti-CD45-FITC | 560975 | BD Pharmigen |
| 488 donkey anti-rabbit IgG | R37118 | Invitrogen |
| 594 donkey anti-mouse IgG | R37115 | Invitrogen |
| Anti-β-Tubulin I antibody | SAB4200715 | Sigma-aldrich |
| Phalloidin-iFluor 488 Conjugate | AAT-23115 | AAT Bioquest |

Antibodies for western-blotting assay.

(I: immunofluorescence; W: western blotting)

| Name | Company | Catalog | Host | Dilution |
| --- | --- | --- | --- | --- |
| OCT3/4 | Santa Cruz | SC-9081 | Rabbit | 1:200(I)/1:1000(W) |
| SOX2 | Millipore | AB5603 | Rabbit | 1:200(I)/1:500(W) |
| NANOG | Cell Signaling | 3580 | Rabbit | 1:200(I)/1:300(W) |
| α-Tubulin | Abcam | Ab11304 | Mouse | 1:10000(W) |
| α-SMA | Abbkine | ABP52852 | Rabbit | 1:300(I) |
| CD63 | Proteintech | 25682-1-AP | Rabbit | 1:300(W) |
| CD81 | Proteintech | 66866-1-lg | Mouse | 1:300(W) |
| CD9 | Proteintech | 20597-1-AP | Rabbit | 1:300(W) |
| T/Brach | Proteintech | 20741-1-AP | Rabbit | 1:300(W) |
| GATA2 | Proteintech | 11103-1-AP | Rabbit | 1:1000(W) |
| PAX6 | Proteintech | 12323-1-AP | Rabbit | 1:500(W) |
| TROP2 | R&D Systems | MAB650-100 | Mouse | 1:500(W) |
| VIM | Proteintech | 10366-1-AP | Rabbit | 1:500(W) |
| FN1 | Proteintech | 15613-1-AP | Rabbit | 1:300(W) |

**Table S3. Chemical compounds.**

| Reagent | Cat. NO. | Source | Concentration/(ng/ml) |
| --- | --- | --- | --- |
| hTPO | 300-18 | PEPROTECH | 20 |
| hSCF | 300-07 | PEPROTECH | 20 |
| hIL-3 | 200-03 | PEPROTECH | 10 |
| hIL-6 | 200-06 | PEPROTECH | 10 |
| hIL-9 | 200-09 | PEPROTECH | 10 |
| hIL-11 | 200-11 | PEPROTECH | 10 |
| Y-27632 | S1049 | SELLECK | 10 |

**Additional References**

1. Zhang L, Liu C, Wang H, Wu D, Su P, Wang M, Guo J, Zhao S, Dong S, Zhou W *et al*: Thrombopoietin knock-in augments platelet generation from human embryonic stem cells. *Stem Cell Res Ther* 2018, 9(1):194.

2. Zhang L, Wang H, Liu C, Wu Q, Su P, Wu D, Guo J, Zhou W, Xu Y, Shi L *et al*: MSX2 Initiates and Accelerates Mesenchymal Stem/Stromal Cell Specification of hPSCs by Regulating TWIST1 and PRAME. *Stem Cell Reports* 2018, 11(2):497-513.

3. Wu Q, Zhang L, Su P, Lei X, Liu X, Wang H, Lu L, Bai Y, Xiong T, Li D *et al*: MSX2 mediates entry of human pluripotent stem cells into mesendoderm by simultaneously suppressing SOX2 and activating NODAL signaling. *Cell Res* 2015, 25(12):1314-1332.
